# Top Information Need Priorities of Older Adults Newly Diagnosed With Active Myeloma

**Published:** 2015-01-01

**Authors:** Joseph D. Tariman1, Ardith Doorenbos2, Karen G. Schepp2, Seema Singhal2, Donna L. Berry2

**Affiliations:** 1School of Nursing/College of Science and Health at De Paul University, Chicago, Illinois; 2University of Washington, Seattle, Washington; 3Dana-Farber Cancer Institute, Boston, Massachusetts

## Abstract

Prioritizing patients’ information needs maximizes efficiency. This study examined the information sources and priorities in a sample of older adults newly diagnosed with symptomatic myeloma requiring immediate therapy. An association analysis of whether information needs were influenced by sociodemographic variables such as age, gender, education, marital status, and income was also conducted. The Information Needs Questionnaire (INQ) and an investigator-developed interview schedule were administered to 20 older adults diagnosed with symptomatic myeloma during a 30- to 45-minute semistructured interview. We found that older adults newly diagnosed with symptomatic myeloma have different priorities of information needs when compared with younger patients diagnosed with various types of cancer. The top three priorities related to treatment, prognosis, and self-care. Sociodemographic variables did not influence the priorities of information needs among older adults with symptomatic myeloma. The Internet, physicians, family, and friends were among the top sources of information. Advanced practitioners in oncology should support and identify interventions that can enhance patients’ learning process from these sources. Well poised to assist patients in searching credible and reliable Internet sources, advanced practitioners in oncology can provide patient education about different treatments and the impact of such treatments on prognosis (e.g., overall survival and likelihood of cure).

Providing pertinent information to patients with cancer is considered an important part of standard care (Jacobson et al., 2009). Patients seek information to understand the diagnosis (and its consequences) and to make treatment and self-care decisions. Evaluating which information needs are of highest priority has been studied in breast, prostate, and other cancer patient populations (Gaston & Mitchell, 2005). In a seminal paper, Degner and colleagues (Degner, Davison, Sloan, & Mueller, 1998) argued that in an era of limited health-care resources, patient information needs are best prioritized. Prioritization of information needs can make patient encounters more meaningful. As reviewed by Husson and colleagues (Husson, Mols, & van de Poll-Franse, 2010), when information is individualized to fulfill what a patient deems to be important, there may be less patient anxiety and depression associated with treatment decision-making.

Oncology advanced practitioners (APs) play a critical role in providing patients the information they need and can potentially help patients assume a more active role in decision-making. Conversely, a lack of information can hinder patients’ participation in treatment decision-making or increase uncertainty. Unfortunately, patients still often report difficulties obtaining the type and amount of information they want or need (Lewis, Gray, Freres, & Hornik, 2009; Mistry, Wilson, Priestman, Damery, & Haque, 2010; Nagler et al., 2010; Walsh et al., 2010). Oncology APs are poised to provide useful, concise, and relevant information, but significant time constraints preclude providing full information required by most patients with cancer (Ousley, Swarz, Milliken, & Ellis, 2010). By determining the particular types of information that patients view as important, APs can potentially identify more quickly what patients want and need to know and assist patients obtain the information they need efficiently.

A new diagnosis of myeloma that requires immediate therapy (active disease, presence of organ involvement or damage such as hypercalcemia, renal insufficiency, moderate anemia, and presence of osteolytic lesions) involves presentation of information about a wide choice of drugs and treatment regimens, including bortezomib (Velcade), lenalidomide (Revlimid), thalidomide (Thalomid), dexamethasone (Mehta, Cavo, & Singhal, 2010; Rome, 2010; Tariman & Faiman, 2011), and autologous or allogeneic hematopoietic stem cell transplantation (Bensinger, 2009; Mehta & Singhal, 2007).

Older adults with cancer have various information needs that are often not met by clinicians (Chouliara, Kearney, Stott, Molassiotis, & Miller, 2004). Because myeloma frequently affects older adults, studying the priority of information needs of these patients could reveal a different one than for younger patients, which has been reported in similar studies conducted in patients diagnosed with breast, colorectal, prostate, lung, gynecologic, and hematologic cancers (Tariman, Doorenbos, Schepp, Singhal, & Berry, 2013).

## PURPOSE

The purpose of this study was to examine the priority information needs of a sample of older adults newly diagnosed with myeloma. The study also examined the common information sources patients used since the time of their diagnosis.

## METHODS

**Design and Sample**

A cross-sectional survey approach involving administration of the Information Needs Questionnaire (INQ) with 36 paired comparisons and an interview schedule during a one-time semistructured interview was employed. The convenience sample consisted of 20 older adults (at least 60 years of age) referred through the Seattle Cancer Care Alliance (SCCA) or the Northwestern University Myeloma Program (NUMP) by several hematologists and oncologists in the greater Seattle or Chicago areas, respectively. Eligibility criteria included adults 60 years of age and older who were (1) newly diagnosed (within the first 6 months) with myeloma requiring immediate therapy, (2) able to read and write English, and (3) able to give informed consent. Non-English speakers were not included in the study because the questionnaire is only available in English and there are limited resources available to the researchers to translate to other languages.

**Procedures and Study Locations**

After obtaining approval from the University of Washington (UW) and Northwestern University (NU) Human Subjects Divisions, eligible participants were recruited to participate in the study. Participants were recruited by mail from both university- and community-based medical practices. At NUMP, the researcher also utilized a direct approach in the recruitment of study participants. A review of clinic schedules at SCCA, NUMP, and UW-affiliated clinics was performed weekly to identify potential study participants.

A total of 79 potential participants were recruited by mail at SCCA from October 2009 through July 2010. Fourteen participants responded and participated in the study (a 17.7% response rate). At NUMP, the researcher identified potential participants through a review of clinic schedule and approached them in person about participation. All six potential participants agreed to participate (a 100% response rate). Informed written consent was obtained from all study participants.

The researcher administered the interview schedule and INQ (Degner et al., 1998) during a semistructured interview conducted in designated research-related conference rooms at SCCA and NUMP. If a participant wanted the interview to be conducted at a later time, a 1-week period following the appointment was allowed for rescheduling. If a participant wanted the interview to be conducted at a different location, that was also facilitated. The participants were informed they could end the interview at any time and could refuse to answer specific questions. The participants were given a $5 Tully’s Coffee gift certificate as reimbursement for their time and effort immediately after the completion of the interview schedule.

**Measures**

The INQ was developed by Degner and colleagues and initially applied in studies of women with breast cancer (Bilodeau & Degner, 1996; Degner et al., 1997). Further use of the tool was extended by Davison in her work with men diagnosed with prostate cancer (Davison, Degner, & Morgan, 1995; Davison, Kirk, Degner, & Hassard, 1999). Subsequently, the tool has been found to be valid and reliable in determining the information needs priorities of patients diagnosed with various malignancies, such as breast, prostate, and colorectal cancers (Beaver, Bogg, & Luker, 1999; Bilodeau & Degner, 1996; Davison et al., 1995; Davison et al., 2002). The commonly used Cronbach’s alpha is not the basis for establishing the reliability of the INQ. As described by Degner and colleagues (1998), the reliability of INQ can be established by calculating the circular triads: Kendall’s consistency (zeta) coefficient and Kendall’s coefficient of agreement and the Mosteller’s chi-square test of internal consistency (Mosteller, 1951).

The INQ contains nine categories of information topics, each with a statement of definition: (1) prognosis (likelihood of cure), (2) stage of disease (spread and extent of cancer), (3) side effects (possible side effects of treatment), (4) treatment options (treatment available), (5) social activities (impact on work, daily activities, and social life), (6) family risk (hereditary risk), (7) home self-care (health-care needs during and following treatment), (8) impact on family (helping family members deal with cancer diagnosis), and (9) sexuality (treatment options and counseling for sexual concerns).

## ANALYSIS

The Thurstone scaling method (Thurstone, 1974) is the traditional analytic approach for the INQ (Degner et al., 1998). Data from the INQ were analyzed using the traditional Thurstone scaling entered into Predictive Analytic Software Statistics version 18 (SPSS Inc., 2009). Sources of information were obtained from the participants’ responses in one of the interview questions.

As described by Degner et al. (1998), the reliability of the INQ in this study was established by the internal rater consistency using the circular triads (a method of measuring complete consistency in the participant’s judgments of which information need category was considered more important than other categories [Edwards, 1974]), Kendall’s consistency (zeta) coefficient, and Kendall’s coefficient of agreement. Mosteller’s chi-square test of internal consistency (Mosteller, 1951) was used to measure internal model consistency. The Mosteller’s chi-square for the present study was 39.39 (*p* = .075), with 28 degrees of freedom, indicating that there is demonstrable consistency between the Case V model and the data in this study (Table 1). A multiple t-test was used to test the significant differences between Thurstone scale scores across the participants. A Bonferroni correction (a method of ensuring that the *p* value must be highly statistically significant for it to be declared a true significant difference) to the *p* value of each subject was applied to correct for multiple testing, as described by Sloan, Doig, and Yeung (1994).

**Table 1:**
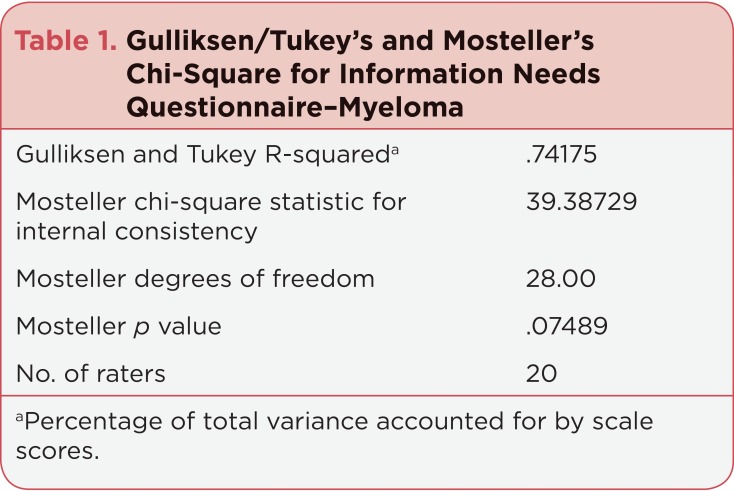
Gulliksen/Tukey’s and Mosteller’s Chi-Square for Information Needs Questionnaire-Myeloma

## FINDINGS

Table 2 presents the sociodemographic characteristics of the 20 study participants. The participants’ mean age was 67.45 years, with a median of 62.5 years. The majority of participants in this sample were Caucasian (90%), female (60%), married (60%), retired (65%), and had at least a 2-year college education (75%). Half of the sample reported an annual household income > $55,000.

**Table 2:**
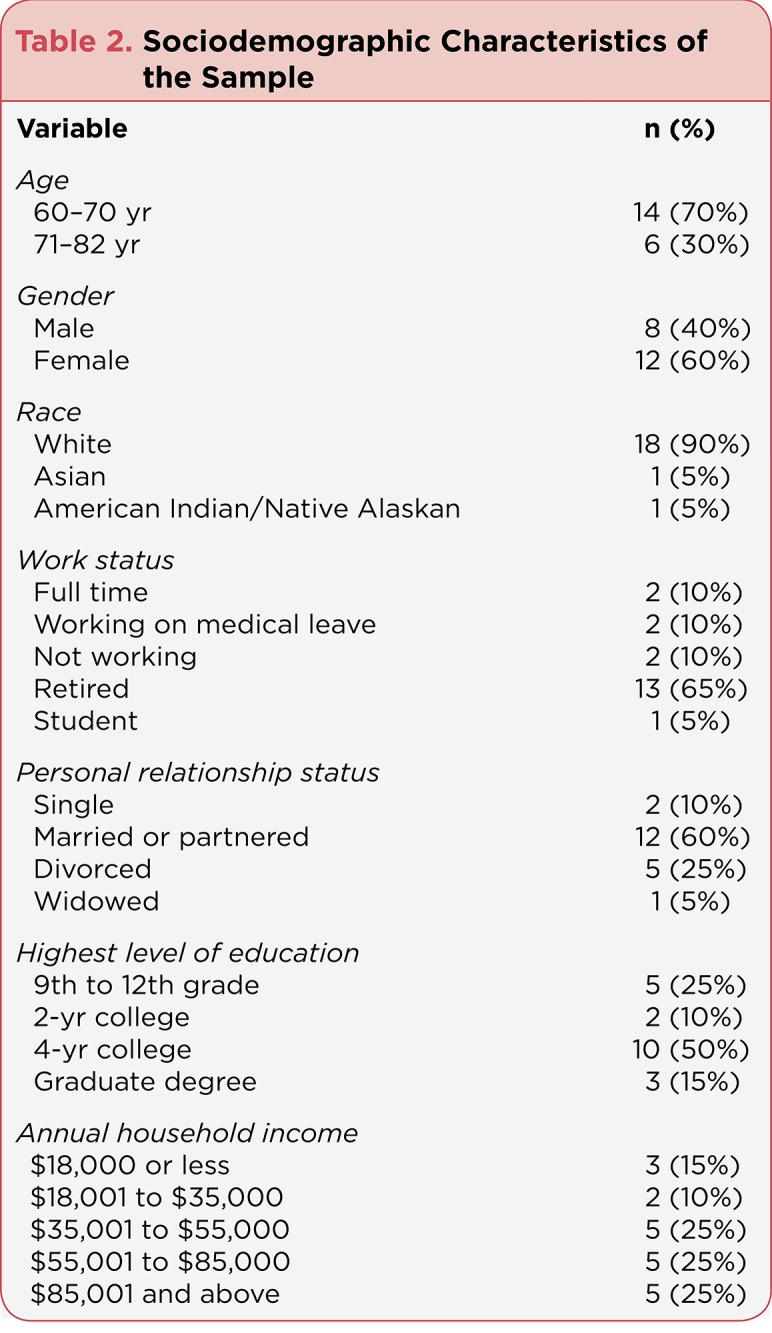
Sociodemographic Characteristics of the Sample

**Priorities of Information Needs**

**(Rank Ordering of Nine Information Items)**

The Thurstone scale analysis for 20 participants newly diagnosed with active myeloma indicated that the top three priority information needs were related to the "different types of treatments" and corresponding advantages/disadvantages, prognosis (likelihood of cure), and self-care or "caring for myself at home" (Figure). The item about the impact of treatment on "feelings about my body and sexual attractiveness" was ranked in last place. Although there is no cure for myeloma, an information category addressing the likelihood of cure was included because of the recent significant improvement in overall survival seen in myeloma patients (Kumar et al., 2008). Depending on the institution’s perspective, cure can be operationally defined as applying to myeloma patients who have continuous periods of remission beyond 5 or 10 years from the initial date of diagnosis.

**Figure 1:**
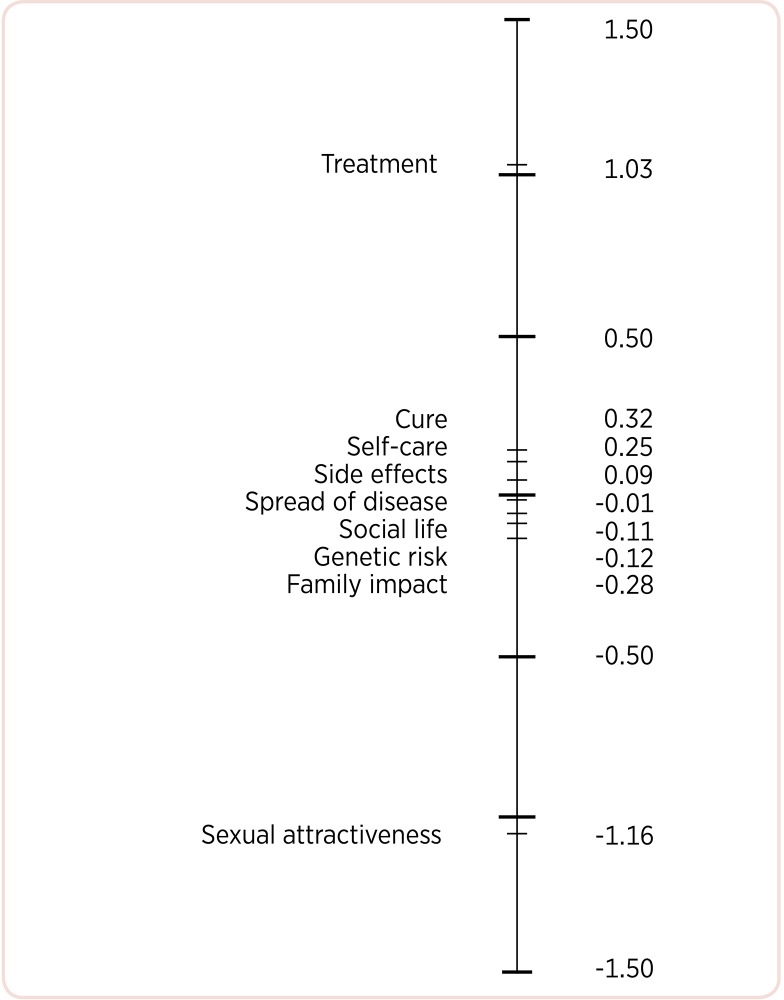
Figure. Priority of information needs by rank. The items on the left are ranked 1 through 9 reading from top to bottom. The items on the right are the Case V scores.

The Thurstone scaling method produced rank orderings or profiles of information needs for older adults newly diagnosed with myeloma. Thus, it overcame the "ceiling effect" of Likert-type scales, in which a majority of participants could rate most information needs as either somewhat or very important (Degner et al., 1998). When asked if there were any other items of information that were important but had not been included in the INQ, two participants provided the following topics of interest: stem cell transplantation logistics (What is the length of the hospital stay?) and overall survival (How long will I live with myeloma?). As these questions seemed critical, consideration of adding them as additional categories in future versions of the INQ merits attention.

**Sources of Information Related to Myeloma**

When patient participants were asked where they had received their information related to myeloma and its therapy, various sources were identified: the Internet (n = 12); physicians, both primary oncologist and second-opinion physicians (n = 7); family and friends (n = 4); books (n = 3); pamphlets (n = 3); nurses (n = 2); and myeloma patients or other cancer patients (n = 3). Some patient participants reported two or three different sources of information.

Analysis of whether information needs were influenced by sociodemographic variables such as age, gender, education, marital status, and income did not reveal any association.

## DISCUSSION

In this study, the Thurstone scaling successfully produced a profile of information needs priorities in older adults newly diagnosed with myeloma that is different from that of younger patients with various types of cancer. This profile suggests that older adults newly diagnosed with myeloma want to discuss the treatment options available to them as well as how these options may impact overall outcomes.

The top two information priorities in myeloma patients are consistent with similar study findings reported in older patients (mean age was 65; age range not reported) with lung cancer (Davidson, Brundage, & Feldman-Stewart, 1999) and older patients (mean ages of breast, prostate, and colorectal cancer patients were 64 [standard deviation (SD) =14], 69 [SD = 9], and 71 [SD = 13] years, respectively) with breast, prostate, and colorectal cancers (Nagler et al., 2010).

It was surprising that disease stage was not one of the top three information priorities in this sample of active myeloma patients. However, as the outcome of myeloma is not heavily influenced by the stage of disease at the time of diagnosis compared with other malignancies, this could explain why the study participants ranked disease stage as the fifth of nine priorities. In myeloma, risk stratification using genomic information has more prognostic significance than disease stage in the new era of novel agents (Kyle & Rajkumar, 2009). It is also a common practice at both research sites (SCCA and NUMP) that patients be informed by physicians that staging has less prognostic significance in myeloma survival than genomics and gene-expression profiling data.

Older adults value independence (Martin & Roberto, 2006), so it was not surprising to find that self-care was one of the top three priority information needs. This finding is supported by another study that found older breast cancer patients (65 years and older) ranked self-care information as more important than did younger patients (Bilodeau & Degner, 1996).

The priorities of information needs reported in this current study contrast with those of younger patients who have been shown to identify prognosis, disease stage, and treatment options as their top priorities in several studies (Tariman, Doorenbos, Schepp, Singhal, & Berry, 2014). Given the fact that treatment options and prognosis are still within the top three information priorities found in this study, one could argue that these two information categories are basic information that are not specific to only older adults with myeloma. However, the priorities of these information categories are definitely different in older adults diagnosed with myeloma than younger patients with various types of cancers, based on the results of the systematic review on information needs priorities in patients with various cancers (Tariman et al., 2014). Moreover, it is unknown whether younger myeloma patients will have different information needs priorities when compared with older adults with myeloma, because no study has been conducted yet to address this question.

**Study Limitations**

Several study limitations must be acknowledged. The rankings in this study must be interpreted with caution, because a single assessment of information needs with a small sample size may not be generalizable. As suggested by Butow and others, periodic assessment of an individual patient’s information needs is imperative, given the changing dynamics of such information needs priorities (Butow, Maclean, Dunn, Tattersall, & Boyer, 1997; Luker, Beaver, Leinster, & Owens, 1996; Vogel, Bengel, & Helmes, 2008). The generalizability of study findings is limited to white men and women who are relatively highly educated and are receiving care at a university-based comprehensive cancer center.

The low response rate (17.7%) to mailed invitations at SCCA is a potential source of response bias. One could argue that patients who did not respond to the mailed flyer may have different profiles of information needs. Moreover, since this is a cross-sectional study, the findings may not be applicable to myeloma patients who are beyond the first 6 months from diagnosis, and these results do not explain any aspect of changes in information priorities over time. A longitudinal approach in a future study should be able to document any changes in the priorities of information needs at different periods of the disease trajectory (i.e., newly diagnosed, first relapse, relapsed and refractory stages).

This study is also limited to myeloma patients who are symptomatic and required immediate therapy. Patients with inactive myeloma (smoldering or indolent myeloma) may have a different information needs profile, and additional studies are needed to compare their information needs with those patients with symptomatic myeloma.

## IMPLICATIONS FOR APs

By profiling information needs in advance, APs may better communicate myeloma-related information to their patients, thereby improving practice and outcomes. A computerized version of INQ has already been tested and was able to efficiently and quickly produce the priority of information needs in patients diagnosed with prostate cancer (Davison, Goldenberg, Gleave, & Degner, 2003; Davison, Goldenberg, Wiens, & Gleave, 2007). The use of this technology, coupled with adequate knowledge and communication skills of the nurse, could potentially offer some innovative solutions to the persistent problem of shrinking time for patient education. Internet-based patient and family member’s education materials could be useful, since the Internet is the leading source of information for patients.

In terms of meeting treatment and prognosis information needs, a team approach is best to help patients make informed decisions.

Oncology APs can provide important information to patients in terms of how the therapy will work and what side effects to expect. They can also share self-care management strategies. As APs and oncology nurses are so frequently in contact with patients, they are in an ideal position to provide additional information related to treatment, prognosis, and self-care. Where information is scant, APs can consult with multidisciplinary team members such as physician residents and fellows, clinical psychologists, and licensed clinical social workers to provide additional information and support. Individualized assessment of information needs at various time points is imperative, given that they often shift over time.

## CONCLUSIONS

This study showed that the top three information priorities in myeloma patients related to treatment, prognosis, and self-care and that they could serve as a starting point to elicit further informational needs. By doing so, APs can eventually improve efficiency in delivering pertinent information to symptomatic myeloma patients. The Internet, physicians, family, and friends are among the top sources of information. Oncology APs should support and identify measures that can enhance patients’ learning processes from these sources. With their graduate education and training, APs are well poised to meet patients’ information needs by assisting them in their search for credible and reliable Internet sources and by educating them about different treatments and their impact on prognosis (e.g., overall survival and likelihood of cure).

**Acknowledgments**

This work was supported by the National Institutes of Health [NIH; NR07106, F31NR011124] and the Achievement Rewards for College Scientists (ARCS) Foundation, Seattle, WA [Pre-doctoral fellowship, 2006-2009]. The content is solely the responsibility of the authors and does not necessarily represent the official views of the NIH and the ARCS Foundation.
